# Small extracellular vesicles secreted by human iPSC-derived MSC enhance angiogenesis through inhibiting STAT3-dependent autophagy in ischemic stroke

**DOI:** 10.1186/s13287-020-01834-0

**Published:** 2020-07-22

**Authors:** Yuguo Xia, Xiaozheng Ling, Guowen Hu, Qingwei Zhu, Juntao Zhang, Qing Li, Bizeng Zhao, Yang Wang, Zhifeng Deng

**Affiliations:** 1 Department of Neurosurgery, Shanghai Jiao Tong University Affiliated Sixth People’s Hospital, No. 600 Yishan Road, Shanghai, 200233 China; 2 Institute of Microsurgery and Extremities, Shanghai Jiao Tong University Affiliated Sixth People’s Hospital, No. 600 Yishan Road, Shanghai, 200233 China

**Keywords:** Human induced pluripotent stem cell-derived mesenchymal stem cells, Small extracellular vesicles, Angiogenesis, Autophagy

## Abstract

**Background:**

Small extracellular vesicles (sEV) secreted by mesenchymal stem cells (MSC) derived from human induced pluripotent stem cells (iPSC, iMSC-sEV) are considered to have great potential in treating ischemic diseases. Angiogenesis play an important role in post-stroke recovery. However, no studies have yet been conducted to systemically examine the effect and the underlying mechanism of iMSC-sEV on angiogenesis under brain ischemia conditions.

**Methods:**

Ischemic stroke model was performed in rats induced by middle cerebral artery occlusion (MCAO), and the pro-angiogenic capacity of iMSC-sEV was measured. The in vitro effects of iMSC-sEV on the migration and tube formation of endothelial cells were investigated, respectively. Autophagy and autophagy-related signaling pathway were detected in vivo and in vitro.

**Results:**

We found that iMSC-sEV significantly reduced infarct volume, enhanced angiogenesis, and alleviated long-term neurological deficits in rats after stroke. We also demonstrated that iMSC-sEV increased migration and tube formation of endothelial cells in vitro. A further mechanism study revealed that the pro-angiogenic effect of iMSC-sEV was correlated with a reduction in autophagy. Furthermore, iMSC-sEV significantly activated signal transducer and activator of transcription 3 (STAT3), and suppression of STAT3 abolished iMSC-sEV-induced inhibition of autophagy and promotion of angiogenesis in vivo and in vitro.

**Conclusions:**

Taken together, our data indicate that iMSC-sEV promote angiogenesis after ischemic stroke, potentially, by inhibiting autophagy, a process that is partially dependent on STAT3 activation.

## Background

Stroke is a leading cause of mortality and disability worldwide [1]. Till now, tissue plasminogen activator (tPA) and endovascular thrombectomy remain the only two effective therapies to treat ischemic stroke [2]. Still, only a small population of patients benefit from the treatments due to the short therapeutic time window or contraindications [3]. Hence, there is an urgent need for effective therapies. Recent experimental and clinical evidences from independent research groups revealed that angiogenesis is correlated with improved neurological recovery after stroke [4–6]. Angiogenesis occurs in the ischemic penumbra within hours after stroke and lasts for weeks. However, ischemic stroke injury stimulated angiogenesis is insufficient to satisfy the need for blood supply and removal of necrotic debris [7]. Hence, developing new and better therapy to augment angiogenesis is of utmost importance for ischemic stroke.

Accumulating studies have reported that cell-based therapy, especially stem cells including embryonic stem cells (ESC), neural stem/precursor cells (NSC/NPC), mesenchymal stem cells (MSC), and induced pluripotent stem cells (iPSC), exerted neuroprotection effect in preclinical stroke research [8]. Among them, MSC are one of the widely studied cells in animal model of ischemic stroke. MSC, with or without modification, have shown remarkable potential of promoting angiogenesis in preclinical ischemic disease studies [9, 10] and clinical trial [11], while using tissue-derived MSC sources including bone marrow, adipose, umbilical cord, muscle, dental pulp, and so on [12] as cell-based therapy has its limitations including invasive procurement procedure (bone marrow biopsy or liposuction), limited cell proliferation potential, age-associated functional decline, and potential risk of rejection for allogeneic cell transfer [12, 13], restricting the clinical applications of MSC. With the advent of reprogramming technique, iPSC have been generated successfully from patients’ adult somatic cells [14]. Indeed, iPSC have attractive features, for example, iPSC possess unlimited self-renewal and differentiation capacity which could provide a large amount of cells [15], and they can generate unlimited early-passage patient-specific MSC with consistent quality [16]. We and other research groups have recently derived MSC from iPSC, providing a new source of MSC (iMSC) [16, 17]. iMSC have been proven to be alike adult MSC in morphology, surface marker expression profile, global gene expression, tri-lineage differentiation capability, and function [18]. Moreover, iMSC overcome the limitations of tissue-derived MSC, which become a promising alternative for stem cell therapy.

Increasing studies have indicated that the efficacy of MSC therapy against stroke might be attributable to the paracrine activity [19, 20] and that small extracellular vesicles (sEV), lipid bilayer nanoparticles containing proteins, lipids, nucleic acid, and other biomolecules, play an important role in this mechanism [20, 21]. sEV are more stable than stem cells under various physiological conditions [22] and can easily cross the blood-brain barrier (BBB) [21], making them suitable for therapeutic interventions for ischemic stroke. Indeed, Doeppner et al. found that MSC-sEV showed a comparable effect on promoting neurogenesis and angiogenesis after stroke with their parental cells [23]. Our group recently demonstrated that iMSC-sEV could attenuate limb ischemia by promoting angiogenesis [24]. However, to date, there is no report on the application of iMSC-sEV to enhance angiogenesis under ischemic stroke conditions.

Recent evidence has suggested that angiogenic behavior of endothelial cells is in a tight relationship with cell autophagy in vitro and in vivo [25]. Autophagy is a dynamic process of subcellular degradation. Study indicated that autophagy protects against ischemic brain injury by removing the accumulated damaged proteins and organelles which can be recycled for cellular defenses and energy generation [26], while autophagy during ischemic stroke is not always protective. Prolonged and excessive autophagy promotes the progressive consumption of cellular constituents and leads to autophagic cell death [27]. Therefore, suppression of detrimental autophagy may be a target for ischemic brain injury. Indeed, autophagy was observed in brain endothelial cells after stroke insult [28, 29]. Some studies suggested that cellular autophagy may inhibit the angiogenesis in endothelial cells [30]. However, the relationship between iMSC-sEV and autophagy in the process of angiogenesis after ischemic stroke remains unclear. In the present study, we investigated the pro-angiogenic effect of iMSC-sEV on a rat model of stroke and further explored the potential mechanism. Here, we show for the first time that iMSC-sEV possess the potential to promote angiogenesis after stroke, at least in part, by inhibiting STAT3-dependent autophagy.

## Materials and methods

### Generation, culture, and identification of iMSC

The use of human iPSC in this study was approved by the local ethics committee of the Shanghai Sixth People’s Hospital affiliated with Shanghai Jiao Tong University. The generation of mesenchymal stem cells from human induced pluripotent stem cells was previously described [16] with a few modifications. The human iPSC line (iPS-S-01) used in this study was from Institute of Biochemistry and Cell Biology of the Chinese Academy of Sciences in agreement with Liao and Xiao [31]. Six-well plate was pre-coated with vitronectin (Nuwacell™ VTN, Nuwacel Biotechnology, RP01002) in a concentration of 1 μg/cm^2^ in DMEM/F12 at room temperature for at least an hour. Next, iPSC were cultured in vitronectin-coated 6-well plate with iPSC culture medium containing basal medium (Nuwacell™ Nova Basal Medium, Nuwacell Biotechnology, RP01001-1) and supplement (Nuwacell™ Nova Supplement, Nuwacell Biotechnology, RP01001-2). When cells reached to 90% confluency, the culture medium was changed to MSC culture medium containing basal medium (Nuwacell™ Nova Missoin Basal Medium, Nuwacell Biotechnology, RP020101) and supplement (Nuwacell™ Nova Missoin Supplement, Nuwacell Biotechnology, RP02010-2). Culture medium was changed every 2 days for 14 days. The cells were then trypsinized with 0.25% trypsin/1 mM EDTA (Gibco) and seeded to 25- and 75-cm^2^ cell culture flasks (Corning) at a density of 1 × 10^5^/mL in MSC culture medium mentioned above. The cells were sub-cultured every 2–3 days when cell reached to 85% confluency. The morphology of cells was changed to fibroblast-like cells at passage 4, and the cells were utilized to identify iMSC phenotypical characteristics and tri-lineage differentiation ability [16]. Passages 5 to 10 were used for the following experiments.

### Multipotent differentiation potential of iMSC

Tri-lineage differentiation capability of iMSC was examined as previously described [16]. Briefly, to detect osteogenesis, iMSC culture medium was switched to osteogenesis medium (Gibco) when 90% confluency was reached. After culture for 21 days, cells were fixed with 4% (w/v) paraformaldehyde (PFA) and Alizarin Red staining was used to detect mineralized calcium. To detect adipogenesis, iMSC were cultured under adipogenesis medium (Gibco) for 21 days, followed by Oil Red O staining. To detect chondrogenesis, 1 × 10^6^ cells were pelleted in a 15-mL polypropylene tube after centrifugation, and chondrogenic medium (Gibco) was gently added to the pellet. After 28 days, the pellet was fixed with 4% (w/v) PFA and embedded in optimum cutting temperature compound (OCT) (Thermo Fisher, Waltham, MA, USA). Cryosections (8 μm) were cut with freezing microtome (Leica, CM1950, Germany) and stained with Toluidine Blue to examine the presence of proteoglycans. The cells cultured in MSC culture medium were served as control. All images were captured under an optical microscope (Leica, DM6B, Germany).

### Flow cytometry analysis

Flow cytometry was used to identify phenotypical markers of iMSC. Single-cell suspension was collected, and cell number was counted. Cells were then incubated with 1% (w/v) bovine serum albumin (BSA) (Gibco) to block the non-specific antigens. Next, 1 × 10^6^ cells were stained with the following conjugated mouse monoclonal antibodies (BD Biosciences): CD73-PE (1:100, 561014), CD29-PE (1:100, 561795), CD44-FITC (1:100, 560977), CD146-PE (1:100, 561013), CD34-APC (1:100, 560940), CD45-FITC (1:100, 560976), CD133-PE (1:100, 566594), and HLA-DR-PE (1:100, 560943). Non-specific fluorescence was determined by incubation of similar cell aliquots with isotype-matched mouse monoclonal antibodies (BD Biosciences). After two washes in 1% (w/v) BSA, the cells were resuspended in 300 μL of 1% BSA and analyzed by CytoFLEX flow cytometer (Beckman Coulter Life Science, USA).

### Isolation and morphological identification of iMSC-sEV

sEV were isolated from the cell culture medium of iMSC by differential ultracentrifugation protocols as previously described [32]. Briefly, the obtained medium was centrifuged at 300*g* for 10 min and 2000*g* for 10 min to remove cells, dead cells respectively. After centrifugation at 10,000*g* for 1 h, the supernatant was filtered through a 0.22-μm filter sterilize Steritop™ (Millipore) to remove cellular debris and microvesicles (MV). The collected medium was further ultracentrifuged at 100,000*g* for 70 min. After removal of the supernatant, the pellet was resuspended in phosphate buffer saline (PBS), followed by another ultracentrifugation at 100,000*g* for 70 min. Finally, pelleted sEV were resuspended in PBS.

### Nano-flow analysis of iMSC-sEV

The size and concentration of the iMSC-sEV were assessed using nano-flow cytometer (N30 Nanoflow Analyzer, NanoFCM Inc., Xiamen, China) as previously described [33]. Briefly, the side scatter intensity (SSI) was measured by the loading of the standard polystyrene nanoparticles (200 nm) with a concentration of 1.58 × 10^8^/mL to the nano-flow cytometer. Next, isolated iMSC-sEV sample diluted with 1000-fold PBS (for a nanoparticle concentration of approximately 5 × 10^9^/mL) was loaded to the nano-flow to measure the SSI. Finally, the concentration of sEV was calculated according to the ratio of SSI to particle concentration in the standard polystyrene nanoparticles. For size measurement, standard silica nanoparticles with mixed size (68 nm, 91 nm, 113 nm, 155 nm) were load to the nano-flow cytometer to generate a standard cure, followed by the loading of sEV sample. The size distribution was calculated according to the standard cure.

### Animal model and iMSC-sEV administration

All animal experiments were approved by the Animal Research Committee of the Shanghai Sixth People’s Hospital (SYXK [Shanghai, China] 2011-0128, 1 January 2011). Male Sprague Dawley (SD) rats (6–8 weeks old, 250–300 g) were randomly assigned to sham or transient middle cerebral artery occlusion (MCAO) groups with different treatments (vehicle or iMSC-sEV tail veil injection) by using a lottery drawing box. Transient focal cerebral ischemia was induced by 2 h MCAO as previously described [34]. Rats in the sham group underwent the same procedure without vascular occlusion. Rectal temperature was monitored and maintained at 37.0 ± 0.5 °C during the entire procedure using a temperature-controlled heating pad. Rats showing no neurological deficits or dead post-MCAO were excluded for data analysis. iMSC-sEV (1 × 10^11^ particles in 500 μL PBS) or vehicle (PBS alone, 500 μL) were administered via tail vein injection 4 h after MCAO based on our previous experience [35].

### In vitro culture of human umbilical vein endothelial cells (HUVECs)

In this study, HUVECs were isolated from human umbilical cords as previously described [16], which were obtained with informed consent and approval by the local ethics committee of the Shanghai Sixth People’s Hospital affiliated to Shanghai Jiao Tong University. Briefly, the cords were washed twice with warm PBS to flush out blood and clots; HUVECs were digested with 0.5 mg/mL type II collagenase (Sigma-Aldrich, St. Louis, MO, USA) for 30 min at 37 °C and drawn out from the vessel wall by medium 200 (M200, Gibco) containing 10% (v/v) fetal bovine serum (FBS, Gibco Life Technologies). After centrifugation at 1000 revolutions per minute (rpm) for 5 min at room temperature, HUVECs were seeded into 1% (w/v) gelatin-coated 25-cm^2^ cell culture flasks in M200 with 10% (v/v) FBS. Once HUVECs reached to 90% confluency, they were further trypsinized and reseeded into 25-cm^2^ cell culture flasks and maintained in M200 supplemented with 2% low serum growth supplement (LSGS, Cascade Biologics, Portland, OR, USA). HUVECs at passage 2 were used in the experiments as described below.

### Uptake of iMSC-sEV in vivo and in vitro

To determine the migration of iMSC-sEV into the brain, iMSC-sEV were stained with DiR (Thermo Fisher, USA) according to the protocol as previously described [36] with small modification. Briefly, sEV were incubated with DiR fluorescent dye under room temperature for 15 min, followed by ultracentrifugation at 100,000*g* in PBS to get rid of the unlabeled dye. Four hours after MCAO procedure, the rats were intravenously administered with a single dose of DiR-labeled sEV (1 × 10^11^ particles in 500 μL PBS). Six hours later, the rats were anesthetized and the DiR fluorescent signals were detected using the IVIS Spectrum imaging system (PerkinElmer, USA). In another experiment, freshly isolated iMSC-sEV were labeled with Dio fluorescent dye (Beyotime biotechnology, China, C1038) according to the manufacturer’s instructions. Briefly, iMSC-sEV were incubated with 10 μM Dio for 30 min under room temperature and washed with PBS twice. Dio-labeled sEV (1 × 10^11^ particles in 500 μL PBS) were administered intravenously 24 h after MCAO and rats were sacrificed 24 h after injection. Brian cryosections (20 μm) were stained with DAPI solution and observed with fluorescence microscope (Leica, DM6B, Germany). In the in vitro experiment, iMSC-sEV were labeled with Dil fluorochrome (Thermo Fisher, USA) according to the manufacturer’s protocol with the same incubation and wash procedures mentioned above. Next, Dil-labeled sEV were added into culture medium and incubated with HUVECs for 4 h. In the control group, the same volume and concentration of Dil dye without iMSC-sEV labeling was ultracentrifuged and washed as mentioned above, followed by incubation with HUVECs. Next, culture medium was discarded, and the cells were rinsed twice with PBS prior image capture under the fluorescence microscope (Leica, DM6B, Germany).

### EdU administration

EdU was utilized to trace cell proliferation after MCAO. Briefly, three doses of 50 mg/kg EdU (Life technologies, USA, e10187) in PBS was intraperitoneally (i.p.) injected in rats at days 3, 5, and 7 after MCAO respectively. Four hours after the last administration of EdU, the rats were sacrificed for later experiments.

### Immunofluorescence staining and quantification

At different timepoint after MCAO as mentioned in specific experiments, the rats were anesthetized and perfused with ice-cold saline, followed by 4% (w/v) paraformaldehyde perfusion. Next, brains were removed, fixed in 4% formaldehyde overnight at 4 °C, and dehydrated with gradient sucrose solutions (20%, 30%, and 35% (w/v)). After being embedded and frozen in an optimal cutting temperature compound (OCT), the brains were sliced into 25-μm-thick coronal sections. The brain sections were then stained with specific markers including CD31 (1:100, Abcam, UK, ab28364), MAP-2 (1:200, Cell Signaling Technology, USA, 4542S), and CD34 (1:100, Abcam, UK, ab81289). Fluorescence images were acquired using a fluorescence microscope (Leica, DM6B, Germany). For the quantification of CD34^+^, CD31^+^/EdU^+^ cell number, and CD31^+^ vascular density, four sections from the region of interest (ROI) were calculated for each rat by ImageJ software (National Institutes of Health, Bethesda, MD, USA). The value from MCAO groups was normalized by the sham group and compared.

### Measurement of brain infarct

Infarct volume was assessed using a 3-Tesla magnetic resonance imaging (MRI) scanner (Siemens, Germany) at day 2 and day 28 and immunofluorescence staining of microtubule-associated protein 2 (MAP-2) at day 28 post-stroke. Images were analyzed using ImageJ software (National Institutes of Health, Bethesda, MD, USA) by an investigator who was blinded to experimental group assignment. Infarct volume was calculated by adding up the consecutive 8 infarct areas (contralateral area minus the non-hyperintense area of the ipsilateral side) with a 1.5-mm interval in the T2-weighted MRI image. The extent of brain infarct was presented as the percentage of the infarct volume versus the corresponding contralateral brain tissue volume.

### Behavior tests

Behavioral tests were conducted for consecutive 3 days before stroke as a training session and 1, 3, 7, 14, 21, and 28 days after stroke using modified neurological severity score (mNSS) [37] and the foot-fault test [34] as described previously. In brief, mNSS was graded as 0 to 18, with a higher score representing more severe neurological dysfunction. Foot-fault was used to test the sensorimotor function. Rats were placed on the elevated grid surface. A foot fault was defined as a step slipping off the grid wire. Each animal was recorded for 3 min per trial per test day. The investigator performing the tests was blinded to group assignment. The data were presented as percentage of foot fault by the contralateral limbs versus total steps.

### Morphology identification of sEV and evaluation of autophagy using TEM

For the observation of morphology in sEV, freshly isolated sEV were loaded onto a continuous carbon grid, fixed in 3% (w/v) glutaraldehyde, and stained with 2% (w/v) uranyl acetate. For autophagy detection, brain tissues or cell pellets were fixed using 3% (w/v) glutaraldehyde for at least 48 h at 4 °C and post-fixed with osmium tetroxide. Samples were dehydrated in a graded series of alcohol concentrations, embedded in epoxy resins, and sectioned. Samples were observed using transmission electron microscopy (TEM; H7650, Hitachi, Tokyo, Japan).

### Oxygen and glucose deprivation (OGD)

HUVECs were seeded onto 6-well plate, and OGD was conducted when the confluency reach to 90%. Briefly, the culture medium of the HUVECs was replaced with glucose-free DMEM (Gibco, NY, USA) containing the same supplements as mentioned above without FBS. The HUVECs were then transferred to anaerobic conditions (5% CO_2_ and 95% N_2_) and incubated for 8 h. OGD was then ended by changing to normal culture medium with FBS and incubated under normoxia conditions (5% CO_2_ and 95% air) with the presence of iMSC-sEV (1 × 10^9^ particles/mL) or vehicle (PBS) for 24 h. Control HUVECs were cultured under normal conditions (5% CO_2_ and 95% air) for the same duration without any treatment. Next, cells were harvested for further analysis.

### Transwell assay

HUVECs were resuspended in culture medium and seeded in the upper transwell chamber with 8 mm pore size (Corning, Lowell, MA, USA), and HUVEC-conditioned medium was added to the lower chamber. After incubation for 24 h, the non-migrated cells on the upper surface of the filter were removed with cotton swab. The migrated cells were fixed with methanol and glacial acetic acid (mixed at 3:1) and then stained using crystal violet staining solution. The migrated cells were imaged using an optical microscope, and the average number of cells was counted in six random fields.

### Tube formation assay

The in vitro tube formation was assayed according to the manufacturer’s instructions. The 200 μl of Matrigel (BD Biosciences, Bedford, MA, USA) solution was added into 48-well plate and incubated at 37 °C for 30 min. HUVECs in a concentration of 1.5 × 10^5^ per mL were seeded on the Matrigel and cultured for 24 h. The total tube length was measured by the ImageJ software.

### STAT3 inhibitor treatment

To inhibit phosphorylation of STAT3 pharmacologically, the static was applied. For the in vivo experiment, stattic with concentration of 3.75 mg per kg was intravenously injected in rats 4 h after MCAO operation accompany with iMSC-sEV. For the in vitro part, HUVECs were challenged by OGD for 8 h and then 5 μM of stattic with iMSC-sEV were added to the culture medium to block the activation of STAT3.

### Western blot analysis

For identification of sEV using western blot analysis, three positive markers of ESC-sEV including CD9, TSG101, and Alix, as well as one negative marker GM130, were evaluated. Specifically, iMSC-sEV were collected as described above. iMSC-sEV proteins were harvested using RIPA lysis buffer (Beyotime biotechnology, China, P0013C) supplemented with protease inhibitor cocktail (Beyotime biotechnology, China, ST505). Next, the protein concentration of iMSC-sEV was measured by the Pierce BCA Protein Assay Kit (Beyotime biotechnology, China, P0012). Proteins were then separated by sodium dodecyl sulfate-polyacrylamide gel electrophoresis (SDS-PAGE, EpiZyme, China) and transferred to polyvinylidene fluoride membranes (PVDF, Millipore, USA). The membranes were blocked with 5% (w/v) non-fat milk for 2 h and incubated overnight under 4 °C with the following antibodies: rabbit monoclonal anti-CD9 (1:1000, Cell Signaling Technology, USA, 13174 s), mouse monoclonal anti-TSG-101 (1:1000, Abcam, UK, ab83), anti-Alix (1:1000, Cell Signaling Technology, USA, 2171 s), and mouse polyclonal anti-GM130 (1:500, Abcam, UK, ab169276). After three washes with TBST, the membranes were incubated with HRP-conjugated secondary antibodies (1:2000, Cell Signaling Technology, USA) under room temperature for 1 h. The immunoreactive bands were visualized using ECL (Thermo Fisher Scientific, USA, WP20005) and imaged with a FluorChem M Fluorescent Imaging System (ProteinSimple, Santa Clara, CA, USA).

For western analysis of HUVECs in the in vitro experiment, cells were seeded on 6-well plate followed by 8-h OGD and 24-h reoxygenation (RO) with or without treatment. Control cells were cultured with the same amount of time as other groups, but without any treatment. After that, cells were washed three times with ice cold PBS, followed by adding of RIPA solution to harvest proteins. Protein concentrations were measured using BCA measurement kit. Total amount of 10-μg protein of cell lysates were run on SDS-PAGE gels. For the in vivo experiment, rats were perfused with ice-cold PBS and the ipsilateral infarct brain tissue was harvest quickly on ice to avoid protein denature, followed by homogenization and lysis in RIPA solution. Total amount of 30-μg protein of tissue lysates were run on SDS-PAGE gels. Next, proteins were transferred to PVDF membrane and then blocked with 5% non-fat milk for 2 h, followed by incubation with primary antibodies against β-actin (1:1000; Abcam, UK, ab133626), LC3 (1:1000; Cell Signaling Technology, USA, 12741), Beclin1 (1:1000; Cell Signaling Technology, USA, 3495), P62 (1:1000; Cell Signaling Technology, USA, 5114), STAT3 (1:1000; Cell Signaling Technology, USA, 9139), and p-STAT3 (1:1000; Cell Signaling Technology, USA, 9145) overnight at 4 °C. After rinse with TBST, membranes were incubated with HRP-conjugated secondary antibodies (1:2000, Cell Signaling Technology, USA) at room temperature for 1 h. Protein level was detected using the ECL detection system. The intensity of each band was analyzed using ImageJ software.

### Statistical analysis

Detailed biological replicates (N) used in each experiment were stated in the figure legend. For western blot (Figs. 4a, d, 5a, c, and 6a, c), tube formation assay (Figs. 3g and 7e and Fig. S5C), and transwell assay (Figs. 3e and 7c and Fig. S5A), each experiment was repeated for at least 3 times using different batch of cells. Data were presented as mean ± SD. The Student *t* test was used to assess the difference between two groups, and the one-way analysis of variance (ANOVA) with the Bonferroni post hoc test was applied for comparisons among multiple groups. Statistical analysis was performed using GraphPad Prism software (version 8.0). Significant difference was considered to be *P* value < 0. 05.

## Results

### Characterization of iMSC and iMSC-sEV

To identify iMSC, firstly, flow cytometry was applied to evaluate the surface antigen profile of the cells. The results showed that iMSC highly express antigen markers including CD73, CD29, CD44, and CD146, but not CD34, CD45, CD133, and HLA-DR (Fig. S1A), which were typical for MSC. Next, the tri-lineage differentiation ability of iMSC was examined. Highly positive cells were visualized in the Alizarin Red (Fig. S1B), Oil Red O (Fig. S1C), and Toluidine Blue staining (Fig. S1D), which indicates the osteogenic, adipogenic, and chondrogenic abilities of iMSC respectively. All these results revealed that the obtained iMSC showed typical characteristics and held multipotent differentiation capability.

**Fig. 1:**
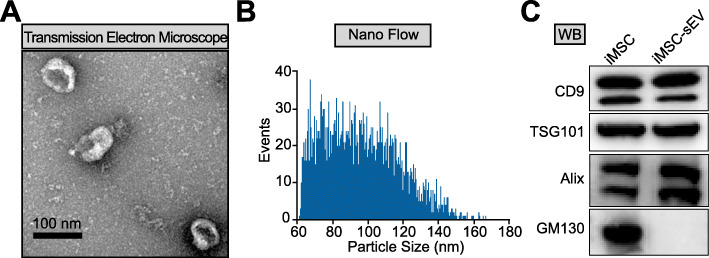
Characterization of iMSC-sEV. **a** Representative image of iMSC-sEV observed by TEM. Scale bar = 100 nm. **b** The particle size distribution of iMSC-sEV measured by nano-flow cytometer. **c** Western blotting showing the expression of exosomal markers including CD9, TSG101, and Alix in iMSC-sEV, but not the negative marker GM130

sEV were isolated from the cell culture supernatant of iMSC and identified using TEM, nano-flow cytometer, and western blot analysis. TEM analysis showed that iMSC-sEV were typical cup-shaped vesicles (Fig. 1a). Nano-flow analysis revealed that the average diameter was ranging from 60 to 160 nm, and the concentration of the iMSC-sEV was approximately 1.36 × 10^11^ particles/mL (Fig. 1b). Western blot analysis determined the presence of exosomal markers, such as CD9, TSG101, and Alix, whereas the cis-Golgi matrix protein GM130 was not detected (Fig. 1c). These data suggested that we had successfully isolated iMSC-sEV.

### iMSC-sEV reduce ischemic brain injury and improve neurological function after MCAO

Firstly, we determined whether intravenous administration of iMSC-sEV could migrate into the brain. iMSC-sEV were labeled with DiR fluorescent dye and administered to rats 4 h after MCAO. While Most of the DiR signals were showed in the lung, liver, spleen, and kidney (data no shown); DiR-labeled iMSC-sEV-treated rats exhibited visible fluorescence in the brain compared to vehicle-treated rats, indicating the ability of iMSC-sEV to cross the blood-brain barrier (BBB) and migrate into the brain (Fig. S2A). Besides, in another set of experiment, Dio-labeled iMSC-sEV were used to track the distribution in the brain. From Fig. S2C, positive fluorescence signals were observed in cells at the peri-infarct area as well as the corresponding contralateral hemisphere, further confirming the previous finding that iMSC-sEV could infiltrate into the brain even with the uncompromised BBB.

**Fig. 2:**
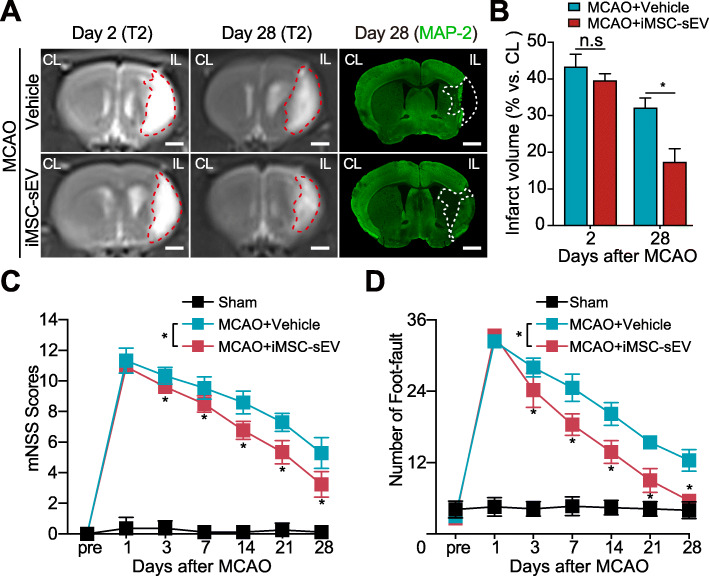
iMSC-sEV treatment reduces tissue loss in the chronic stage of ischemic stroke and promotes long-term neurological recovery. **a** Representative images of T2-MRI scan (left two columns) performed on days 2 and 28, as well as MAP-2 immunostaining (Right column) on day 28 after MCAO. *Dashed line (red and white)*: infarct border. CL, contralateral side. IL, ipsilateral side. Scale bar = 2 mm. **b** Quantification analysis of the percentage of infarct volume on day 2 and day 28 after MCAO. *N* = 8 rats per group. Behavior tests were performed by using mNSS score (**c**) and foot-fault test (**d**) before and up to 28 days after MCAO. *N* = 5 rats for sham group. *N* = 10 rats for vehicle and iMSC-sEV groups. Data are presented as mean ± SD. *P < 0.05. n.s indicates no significant difference

We then investigated the therapeutic effects of iMSC-sEV in rats after ischemic stroke. Based on our previous experience in treating rat model of ischemic stroke using urinal stem cell derived sEV (USC-sEV) [35], the dose of 1 × 10^11^ iMSC-sEV particles number was chosen for the following in vivo experiment. Brain infarct size was monitored using MRI on day 2 and day 28 and MAP-2 staining on day 28 after MCAO (Fig. 2a, b). No significant difference was visualized for infarct volume between two groups on day 2 after stroke (Fig. 2a, b). However, iMSC-sEV treatment significantly attenuated brain infarct as compared to the vehicle group 28 days after MCAO (Fig. 2a, b). Measurements from behavioral tests were applied to examine whether iMSC-sEV contribute to long-term improvement in neurological function after stroke. As expected, compared with vehicle treatment, iMSC-sEV significantly enhanced functional recovery, as reflected by significantly decreased mNSS (Fig. 2c) and lower error step number in the foot-fault test (Fig. 2d), starting from early 3 days to 28 days after stroke. These results indicated that iMSC-sEV improve functional outcomes in rats after cerebral ischemic injury.

### iMSC-sEV enhance angiogenesis after ischemic stroke

**Fig. 3:**
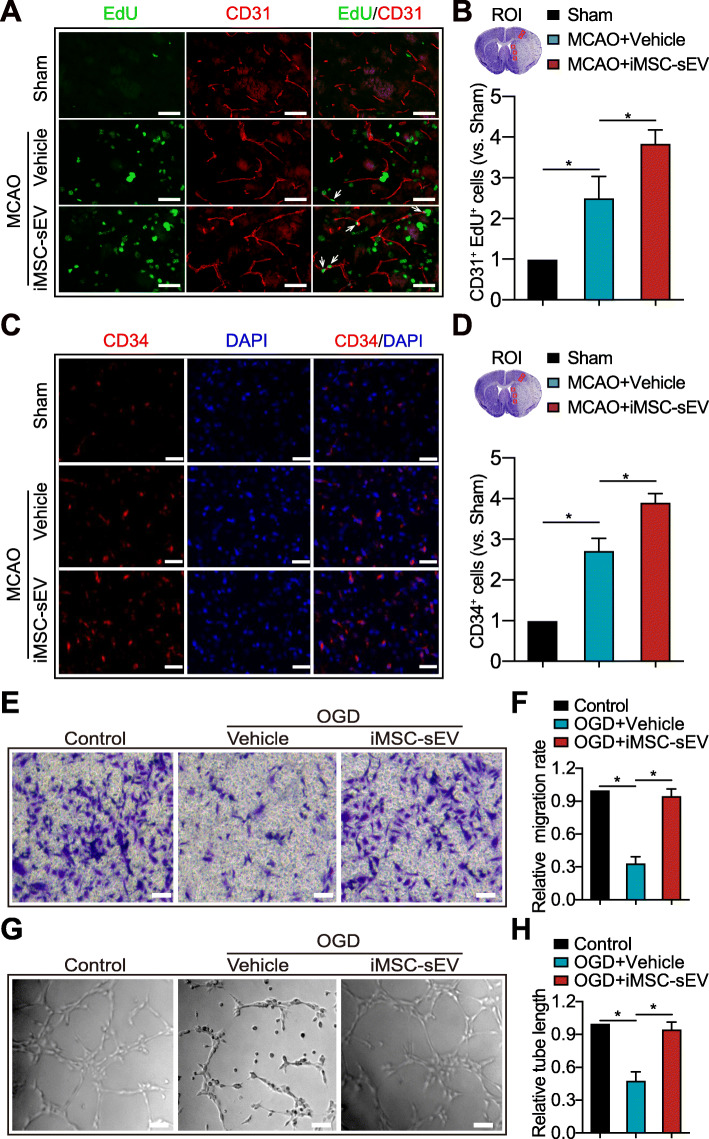
iMSC-sEV promote angiogenesis after stroke and increase migration and tube formation in HUVECs after OGD. **a**–**d** Angiogenesis was assessed by immunofluorescence staining of CD31/EdU and CD34 at 7 days after MCAO. **a** Representative images of CD31 (red) and EdU (Green) in the ischemic boundary zone. *Arrow indicates CD31*^*+*^*EdU*^*+*^*proliferated endothelial cells.* Scale bar = 100 μm. **b** Nissl staining (upper left) showing the region of interest (ROI) and quantification analysis of CD31^+^EdU^+^ cells normalized to that in sham group. *Red box:* ROI of CD31/EdU staining. *N* = 3–5 per group. **c** Representative images of CD34 (red) and DAPI (blue) in the peri-infarct area. Scale bar = 100 μm. **d** Nissl staining (upper left) showing ROI of the staining and quantification analysis of CD34^+^ cells normalized to that in sham group. *Red box:* ROI of CD34 staining. *N* = 3–5 per group. **e**–**h** HUVECs were challenged with 8 h OGD, followed by iMSC-sEV or vehicle treatment for 24 h. HUVECs cultured under the normoxia condition without treatment were set as control. **e** Representative images of crystal violet staining in the transwell assay. Scale bar = 25 μm. **f** Quantification analysis of migration rate normalized to that in control group. *N* = 3 per group. **g** Representative images of the tube formation assay. Scale bar = 25 μm. **h** Quantification analysis of the relative tube length normalized to that in control group. *N* = 3 per group. Data are presented as mean ± SD. *P < 0.05

Angiogenesis is positively correlated with neurological function recovery after stroke [6]. Next, we examined if iMSC-sEV could promote angiogenesis after ischemic stroke in rats. Newly formed vessels and mature vessels in the infarct boundary zone were characterized by CD31/EdU, CD34 staining, and CD31 staining [38]. The vehicle group induced a significantly higher expression of CD31^+^/EdU^+^ (Fig. 3a, b) and CD34^+^ endothelial cells (Fig. 3c, d) than the sham group, suggesting a self-protecting mechanism of spontaneous angiogenesis after stroke insult. Notably, a further increased number of CD31^+^/EdU^+^ (Fig. 3a, b) and CD34^+^ cells (Fig. 3c, d) were detected in the iMSC-sEV group as compared to the vehicle group. In addition, comparing to PBS-treated group, the overall blood vessel density was increased in the iMSC-sEV treatment group 7 days after MCAO (Fig. S3A-B). Taken together, these data suggested that post-stroke iMSC-sEV administration promotes angiogenesis.

### iMSC-sEV promote migration and tube formation of HUVECs subjected to OGD

To further determine the effect of iMSC-sEV on the function of endothelial cells, HUVECs were applied to oxygen and glucose deprivation (OGD), a well-established in vitro model to mimic ischemic stroke. First of all, HUVECs were identified by the expression of CD31 (Fig. S4A) and vWF (Fig. S4B) but not α-SMA (Fig. S4C). We then determined whether iMSC-sEV could be internalized by HUVECs. iMSC-sEV were labeled with Dil fluorescent dye and added to HUVECs culture medium. After 4 h of incubation, Dil-labeled iMSC-sEV were efficiently up-taken by HUVECs (Fig. S4D). HUVECs subjected to OGD exhibited significantly decreased abilities of migration and tube formation as compared to control group, as shown in Fig. 3e–h iMSC-sEV significantly enhanced the migration capability of HUVECs when compared to the vehicle treatment. Besides, an improved tube forming ability was also visualized in the iMSC-sEV group, compared to that in the vehicle group 24 h after administration. Collectively, these data indicated that iMSC-sEV promote migration and tube formation of HUVECs subjected to OGD in vitro.

### iMSC-sEV alleviate ischemic stroke-induced autophagy in vivo and in vitro

**Fig. 4:**
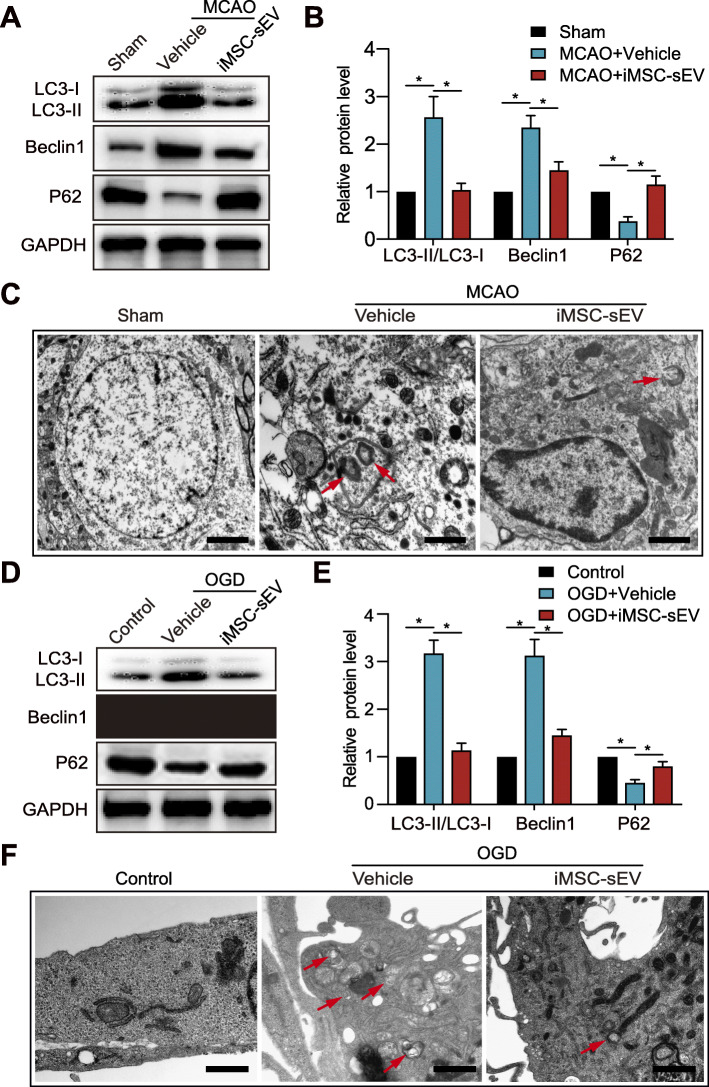
iMSC-sEV treatment alleviates stroke-induced autophagy in vivo and in vitro. Ipsilateral infarct brain tissue was harvested 24 h after MCAO or Sham operation for western blotting analysis (**a**–**b**) or TEM observation (**c**). **a**, **b** Protein expression levels of autophagy associated proteins including LC3-II/LC3-I, Beclin-1, and P62 in the indicated groups. *N* = 3–5 per group. **c** Representative images of TEM showing autophagy-related morphological change after MCAO. *Red arrow:* autophagosome. Scale bar = 1 μm. **d–f** HUVECs were cultured in OGD condition for 8 h, followed by normoxia condition with the treatment of iMSC-sEV or vehicle for another 24 h. HUVECs cultured under normoxia condition without treatment were used as control. **d**, **e** Western blotting was used to evaluate LC3-II/LC3-I, Beclin-1, and P62 protein levels in HUVECs. *N* = 3 per group. **f** Representative TEM images of HUVECs with or without OGD challenge. *Red arrow:* autophagosome. Scale bar = 1 μm. Data are presented as mean ± SD. *P < 0.05

Autophagy occurred in endothelial cells in a rat model of MCAO [28]. We then investigated the relationship between autophagy and the pro-angiogenic effects of iMSC-sEV in ischemic stroke models in vivo and in vitro. We first detected the autophagy-associated protein levels after MCAO by western blot. The results showed that the expression of LC3-II/LC3-I and Beclin-1 was significantly increased while P62 protein level was decreased after stroke model (Fig. 4a, b). However, iMSC-sEV markedly reduced the protein levels of LC3-II/LC3-I and Beclin-1 and increased P62 level. We also used TEM to detect autophagy in vivo. The specific autophagosomes which is characterized by double-membrane structure was visualized in the vehicle group but not the sham group (Fig. 4c). Notably, the iMSC-sEV group showed less autophagosome.

Next, to detect autophagy in endothelial cells after stroke, HUVECs were subjected to an 8-h OGD. In consistent with the in vivo data, results from western blot demonstrated that OGD increased levels of LC3-II/LC3-I and Beclin-1 while decreased p62 protein levels in HUVECs compared to control group, and iMSC-sEV dramatically suppressed OGD-induced LC3-II/LC3-I and Beclin-1 expression and increased p62 expression in HUVECs (Fig. 4d, e). TEM indicated that iMSC-sEV reduced the formation of autophagosomes during OGD in HUVECs (Fig. 4f). These results indicated that iMSC-sEV inhibit ischemic stroke-triggered autophagy.

We have proved iMSC-sEV treatment promoted angiogenesis and inhibited autophagy in endothelial cells after stroke. To further prove the interaction between autophagy and angiogenesis in stroke conditions, 3-methyladenine (3-MA), a well-known autophagy inhibitor, was applied. As expected, 3-MA reversed the decline of migration (Fig. S5A-B) and tube formation (Fig. S5C-D) induced by OGD in HUVECs, suggesting that increase of angiogenesis is related to the inhibition of autophagy in HUVECs after stroke.

### iMSC-sEV activated STAT3 signaling pathway in vivo and in vitro

**Fig. 5:**
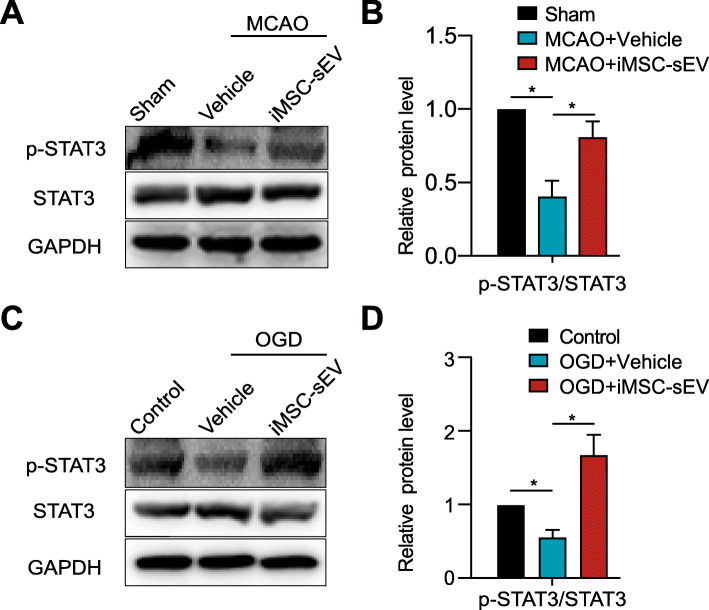
iMSC-sEV activate STAT3 signaling pathway in vivo and in vitro. **a**, **b** Western blotting analysis showing the expression level of p-STAT3 and STAT3 in the ipsilateral infarct brain 24 h after MCAO or sham operation. *N* = 3–5 per group. **c**, **d** HUVECs were cultured in OGD condition for 8 h, followed by normoxia condition with the treatment of iMSC-sEV or vehicle for another 24 h. Western blotting analysis was performed to evaluate the expression of p-STAT3 and STAT3 in HUVECs in indicated groups. *N* = 3 per group. Data are presented as mean ± SD. *P < 0.05

Many signaling pathways mediate autophagy, and STAT3 has been considered as a classical inhibiting factor [39]. Therefore, STAT3 was detected in vivo and in vitro using western blot analysis. Phosphorylated STAT3 decreased significantly in ischemic stroke rats compared to sham rats, and iMSC-sEV dramatically reversed this decrease (Fig. 5a). A similar result was confirmed in vitro (Fig. 5b). Altogether, these results suggested that iMSC-sEV activate the STAT3 signaling pathway.

### STAT3 signaling pathway is involved in iMSC-sEV’s inhibition of stroke-induced autophagy

**Fig. 6:**
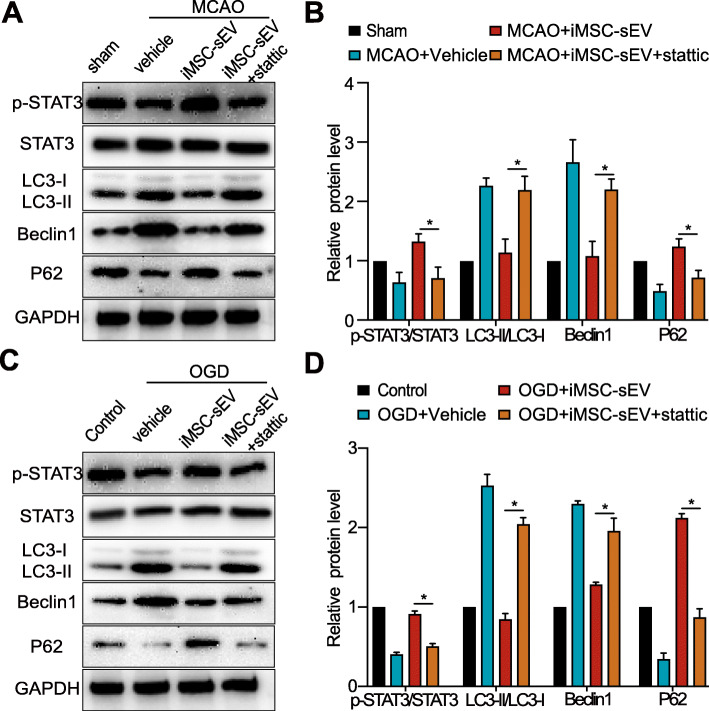
iMSC-sEV inhibit stroke-induced autophagy partially via activation of STAT3 signaling pathway. **a**, **b** Rats were intravenously treated with vehicle (500 μl, PBS), iMSC-sEV (1 × 10^11^ particles in 500 μL PBS), or iMSC-sEV with stattic (3.75 mg/kg) 4 h after MCAO, and infarct brain tissue from different groups was harvested 24 h after MCAO for western blotting. Sham rats were used as control. Representative images (**a**) and quantification (**b**) of the proteins including p-STAT3/STAT3, LC3-II/LC3-I, Beclin-1, and P62 in the indicated groups. *N* = 3–5 per group. **c**, **d** HUVECs were challenged by OGD for 8 h, followed by treatment with vehicle (PBS), iMSC-sEV (1 × 10^9^ particles/mL), or iMSC-sEV with stattic (5 μM) under normal culture condition for another 24 h. HUVECs cultured under normoxia condition without treatment were used as control. Representative bands (**c**) and quantification (**d**) of the proteins including p-STAT3/STAT3, LC3-II/LC3-I, Beclin-1, and P62 in the indicated groups. *N* = 3 per group. Data are presented as mean ± SD. *P < 0.05

iMSC-sEV significantly activated STAT3 after ischemic stroke in vivo and in vitro. We next used stattic, a STAT3 inhibitor, to further confirm the role of STAT3 in the preventative effects of iMSC-sEV on stroke-induced autophagy. Western blot analysis showed that stattic significantly decreased p-STAT3/STAT3 ratio in the iMSC-sEV supplemented with stattic group as compared to iMSC-sEV group both in vivo (Fig. 6a, b) and in vitro (Fig. 6c, d), suggesting stattic inhibited iMSC-sEV-induced STAT3 activation. Accompanied by this phenomenon, stattic also abolished the iMSC-sEV-induced reduction of Beclin-1and LC3-II/LC3-I, and increment of p62 both in vivo (Fig. 6a, b) and in vitro (Fig. 6c, d). These results suggested that the STAT3 signaling pathway takes part in iMSC-sEV’s suppression of stroke-induced autophagy after stroke.

### iMSC-sEV facilitate angiogenesis partially via upregulation of STAT3 in vivo and in vitro

**Fig. 7:**
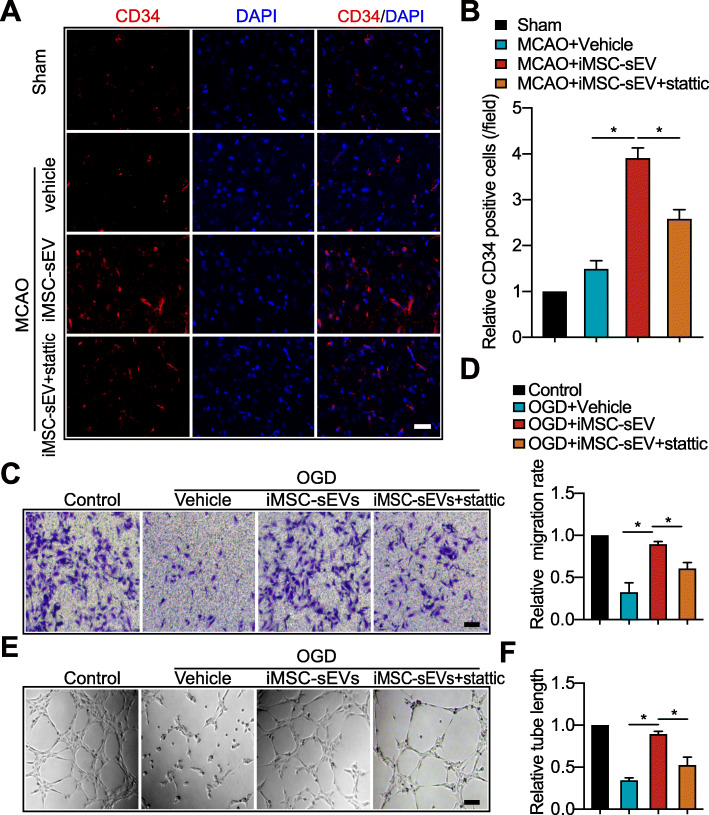
Inactivation of STAT3, partly, inhibits the pro-angiogenic effect of iMSC-sEV after the experimental stroke model. **a**, **b** Rats were intravenously treated with vehicle (500 μl, PBS), iMSC-sEV (1 × 10^11^ particles in 500 μL PBS), or iMSC-sEV with stattic (3.75 mg/kg) 4 h after MCAO; angiogenic ability was assessed 7 days after MCAO or Sham surgery. **a** Representative images of immunofluorescence staining of CD34 in the peri-infarct area. Scale bar = 100 μm. **b** Quantification analysis of CD34^+^ cells normalized to that in sham group. *N* = 3 per group. **c**–**f** HUVECs were challenged by OGD for 8 h, followed by treatment with vehicle (PBS), iMSC-sEV (1 × 10^9^ particles/mL), or iMSC-sEV with stattic (5 μM) in normal culture medium for another 24 h. HUVECs cultured under normoxia condition without any treatment were deemed as control. **c** Representative images of crystal violet staining of HUVECs in the transwell assay. Scale bar = 25 μm. **d** Quantification analysis of relative migration rate. *N* = 3 per group. **e** Representative images of the tube formation assay. Scale bar = 5 μm. **f** Quantification analysis of relative tube length in the indicated groups. *N* = 3 per group. Data are presented as mean ± SD. *P < 0.05

The STAT3 inhibitor stattic was used to examine whether the pro-angiogenic effect of iMSC-sEV on ischemic stroke was related to STAT3-dependent autophagy. Stattic significantly abolished the iMSC-sEV-induced increase of CD34^+^ new-born endothelial cells (Fig. 7a, b), CD31^+^EdU^+^ proliferated endothelial cells (Fig. S6A-B), and CD31^+^ vessel density (Fig. S6C-6D) in the infarct boundary zone 7 days after MCAO. However, the diminishment induced by STAT3 blocking did not reach to the level of vehicle group, indicating another pathway may be involved in the promotion effect of iMSC-sEV on angiogenesis (Fig. 7a, b). Besides, in vitro study also showed that stattic reversed the promotion of migration and tube formation by iMSC-sEV in HUVECs after OGD (Fig. 7c–f). Taken together, these data indicated that STAT3 play an important role in the pro-angiogenic effect by iMSC-sEV in ischemic stroke.

## Discussion

In this study, we found that iMSC-sEV reduced infarction size and improved neurological recovery in an experimental ischemic stroke model in rats. We further demonstrated that iMSC-sEV significantly increased newly formed blood vessels and mature vessels after stroke. Moreover, iMSC-sEV promoted both migration and tube formation in HUVECs subjected to OGD. Mechanistically, iMSC-sEV promoted angiogenesis in the ischemic brain, in part, via suppression of autophagy, a process that is dependent on STAT3 activation. The present study is the first to report that iMSC-sEV promote angiogenesis and protect against ischemic brain injury, potentially, via the inhibition of autophagy, and STAT3 pathway played an important role in this process.

Angiogenesis is a complicated and sequential process which plays a crucial role in ischemic brain injury. The newly formed vessels after stroke not only improved tissue perfusion but also closely linked with neurovascular remodeling [40], axonal sprouting [41], and remyelination [42]. It is well established that angiogenesis is strongly associated with improvement of neurological deficits after stroke [5, 6]. Patients with higher blood vessel density showed better functional recovery after ischemic stroke impact. The ability to promote angiogenesis in MSC from different sources, for example, bone marrow [43], human umbilical blood [44], and adipose [45], were widely studied. However, current methods for the large-scale preparation of MSC face several challenges as the amount of MSC that may be obtained from donors is often insufficient. Furthermore, the potential of growth and differentiation in vitro is affected by various factors such as culture period, age, and health condition of the donor. Given that iMSC provide an ideal method that can avoid ethical problem and immune rejection, our strategy for sEV production offers several advantages considering the limitations related to the present applications of MSC. Previous studies from our group demonstrated that iMSC-sEV had a strong therapeutic function by promoting angiogenesis in different disease models including limb ischemia [16] and osteonecrosis [46]. Thus, we proposed that iMSC-sEV may also have a similar function in ischemic stroke. Consistent with our hypothesis, rats treated with iMSC-sEV presented with decreased brain tissue loss, improved neurological outcome, and higher vessel density after stroke, suggesting that iMSC-sEV attenuate ischemic brain injury partially through promotion of angiogenesis. We further found that in vitro ischemia model, OGD, induced markedly decline in endothelial functions (migration and tube formation) which was rescued by iMSC-sEV treatment, confirming the ability of iMSC-sEV in promoting angiogenesis under stroke conditions.

Autophagy is activated after brain ischemia, and it is detected in different brain cells including neurons, oligodendrocytes, endothelial cells, and so on^26^. The effect of autophagy on ischemic injury remains controversial with some studies reported a protective role while others showed a deleterious function [47]. For example, activation of autophagy by rapamycin, a mTOR inhibitor, reduced infarction and improved outcome in murine models of MCAO [48]. In contrast, Shi et al. reported that excessive autophagy contributes to neuronal death [27]. Additionally, reducing autophagy both in vitro and in vivo is beneficial during ischemic stroke [49, 50]. In our study, we found that autophagy is markedly activated in infarct penumbra 24 h after stroke or in OGD-treated HUVECs. iMSC-sEV administration downregulated the protein expression of Beclin1 and the LC3-II/I ratio, both of which are markers for autophagy, and upregulated the expression of p62 in vivo and in vitro. The controversial action of autophagy in ischemic stroke studies may be due to the complexity of experimental settings including differences in animal model and stage and intensity of ischemia [51]. Indeed, we conducted a 2-h MCAO model in rats, which maybe induced excessive and prolonged autophagy which is detrimental after stroke. Previous studies reported a complicated crosslink between angiogenesis and autophagy. Some studies have shown that cellular autophagy may enhance angiogenesis in endothelial cells [52, 53]. On the contrary, increasing evidence suggested that cellular autophagy can inhibit the angiogenesis in endothelial cells [54–56]. Consistent with previous studies which demonstrated that MSC-sEV can regulate autophagy [57], our results showed that ischemia-induced autophagy suppressed angiogenesis in vivo and in vitro, while inhibition of autophagy by iMSC-sEV significantly promoted angiogenesis in vivo and facilitated cell migration and tube formation in OGD-treated HUVECs in vitro. These data suggested that iMSC-sEV may increase angiogenesis by inhibiting endothelial autophagy during the recovery of ischemic stroke.

The reasons underlying the inhibitory effect of MSC-sEV against autophagy under ischemic conditions may be involved with the transfer of substances contained in sEV including microRNAs [58–61] and functional proteins [62]. For example, miR-20a containing sEV derived from umbilical cord mesenchymal stem cells (UC-MSCs) inhibited autophagy by binding to 3′ UTR of Beclin-I and alleviated liver ischemia/reperfusion injury [59]. In addition, in an animal model of myocardial infarction (MI), treatment with MSC-derived sEV overexpressing miR-125b reduced autophagic flux and infarct size, along with improved cardio functions [61]. Upregulation of stromal-derived factor 1 (SDF1a) with SDF1 plasmid in MSC-derived sEV inhibited ischemia-induced autophagy and promoted cardiac endothelial microvascular regeneration after MI insult [62].

Many signaling pathways mediate autophagy, and STAT3 is a classical inhibiting factor [39]. The present study detected that ischemic stroke decreased STAT3 activation in vivo and in vitro. iMSC-sEV significantly upregulated the STAT3 signaling pathway in the peri-infarct area in rats after stroke and cultured HUVECs subjected to OGD in the present study. Moreover, blocking STAT3 activation partially abolished iMSC-sEV’s inhibition of stroke-induced autophagy and angiogenesis. Previous studies demonstrated that STAT3 reduced Beclin-1 expression, thus suppressing autophagy via inhibition of oxidative stress and autophagy-related signaling molecules such as FOXO1 and FOXO3 [39]. Therefore, STAT3 may inhibit autophagy via a reduction in Beclin-1 in ischemic stroke. The results of the present study demonstrated that iMSC-sEV increased STAT3 activation during ischemic stroke, which was accompanied by a reduction in Beclin-1. However, STAT3 inhibitor abolished the iMSC-sEV-induced inhibition of Beclin-1. These results indicated that iMSC-sEV inhibits ischemic stroke-provoked autophagy via a STAT3-dependent pathway. The role of STAT3 signaling pathway on brain vessel after stroke is multifaceted. Conditional knockout of endothelial STAT3 reduced angiogenesis and exacerbated neurological deficits after stroke, suggesting an important role of STAT3 in regulating angiogenesis [63]. This result is consistent with our study where increased activation of STAT3 by iMSC-sEV promoted angiogenesis and inactivation of STAT3 by stattic partially attenuated this effect, indicating a potential STAT3-involved mechanism in iMSC-sEV-induced pro-angiogenic ability after ischemic brain injury, while, a recent paper reported that inhibition of STAT3 by stattic improved blood brain barrier (BBB) integrity after stroke [64]. Indeed, the effect of STAT3 activation on other aspects such as neuronal death and neuroinflammation after stroke is still in debate [65]. Further investigation to elucidate the thorough functions of STAT3 after stroke is in need.

## Conclusion

In summary, the present study reported, for the first time, that iMSC-sEV treatment protects against ischemic injury and promotes angiogenesis probably via the inhibition of autophagy. The mechanism for iMSC-sEV’s inhibition of autophagy was partially due to the activation of STAT3. This finding provides a novel treatment strategy for ischemic stroke and uncovers new molecular mechanisms underlying the application of iMSC-sEV.

## Supplementary information

**Additional file 1: Figure S1.** Phenotypic characteristics of iMSC. **(A)** Flow cytometry analysis of the surface antigen profile of iMSC. **(B-D)** Representative images of Alizarin Red staining **(B)**, Oil Red O staining **(C)**, and Toluidine Blue staining **(D)** for the evaluation of osteogenesis, adipogenesis, and chondrogenesis in iMSC. Scale bar = 25 μm.

**Additional file 2: Figure S2.** In vivo uptake of iMSC-sEV after stroke. **(A)** One single dose of vehicle (PBS, 500 μL) or DiR labeled iMSC-sEV (DiR-iMSC-sEV, 1 × 10^11^ particles in 500 μL PBS) were administered through tail veil injection in rats 4 h after MCAO and images were captured 6 h after administration. Representative fluorescence images of rats brain in the vehicle and DiR-iMSC-sEV group. *IL:* ipsilateral side. *CL:* contralateral side. **(B-C)** Dio labeled iMSC-sEV (Dio-iMSC-sEV) were administered intravenously 24 h after MCAO, and rats were sacrificed 24 h after injection. **(B)** Illustration of experimental design. **(C)** Representative images of Dio-iMSC-sEV (green) in the ipsilateral and contralateral side of the brain. *Yellow dashed line: outline for blood vessel. Red arrow head: Dio-iMSC-sEV around the nucleus in the cytoplasm.* Scale bar = 50 μm.

**Additional file 3: Figure S3.** Treatment of iMSC-sEV increases the blood vessel density after ischemic stroke. CD31 immunofluorescence staining was utilized to evaluate blood vessel density 7 days after MCAO. **(A)** Representative images of CD31 positive endothelial cells and DAPI staining in the peri-infarct area. Scale bar = 400 μm. **(B)** ROI and quantification of CD31^+^ blood vessel density. *N* = 3–5 per group. Data are presented as mean ± SD. *P<0.05.

**Additional file 4: Figure S4.** Identification of HUVECs and in vitro uptake of iMSC-sEV by HUVECs. **(A-C)** Representative immunofluorescence images of CD31 (green), vWF (green), and α-SMA (red) in HUVECs. Scale bar =100 μm. **(D)** Representative immunofluorescence images of HUVECs cultured with Dil labeled iMSC-sEV (red) or Dil alone (control). The Dil-labeled iMSC-sEV were visible in the perinuclear region of recipient cells. HUVECs in the control group showed no fluorescence signal. Scale bar =30 μm.

**Additional file 5: Figure S5.** Inhibition of autophagy decreases migration and tube formation in HUVECs after OGD. HUVECs were challenged with 8 h OGD with the addition of 3-MA (5 mM) or vehicle (PBS) and migration and tube formation were analyzed 24 h after reoxygenation. HUVECs cultured under the normoxia condition without treatment were set as control. **(A)** Representative images of crystal violet staining in the transwell assay. Scale bar = 25 μm. **(B)** Quantification analysis of migration rate normalized to control group. N = 3–5 per group. **(G)** Representative images of the tube formation assay. Scale bar = 25 μm. **(H)** Quantification analysis of the tube length normalized to control group. N = 3–5 per group. Data are presented as mean ± SD. *P<0.05.

**Additional file 6: Figure S6.** Stat3 inactivation inhibites iMSC-sEV-induced angiogenesis. **(A-D)** Rats were intravenously treated with vehicle (500ul, PBS), iMSC-sEV (1 × 10^11^ particles in 500 μL PBS), or iMSC-sEV with stattic (3.75 mg/kg) 4 h after MCAO, EdU was i.p. injected at day 3, 5, and 7 to label proliferated cells. **(A)** Representative images of CD31^+^EdU^+^ endothelial cells in the peri-infarct area. *Arrow head, double-labeled proliferated cells*. Scale bar = 100 μm. **(B)** Quantification of CD31^+^EdU^+^ endothelial cells normalized to that in sham group. N = 3–5 per group. **(C)** Representative of CD31^+^DAPI^+^ endothelial cells in the peri-infarct zone. Scale bar = 400 μm. **(D)** Quantification of CD31^+^ vascular density normalized to that in Sham group. N = 3–5 per group.

## Data Availability

The datasets used and/or analyzed during this study are available from the corresponding author on reasonable request.

## Abbreviations

iPSC: Human induced pluripotent stem cells
MSC: Mesenchymal stem cells
STAT3: Signal transducer and activator of transcription 3
sEV: Small extracellular vesicles
MCAO: Middle cerebral artery occlusion
iMSC-sEV: Small extracellular vesicles secreted by mesenchymal stem cell derived from human induced pluripotent stem cells
tPA: Tissue plasminogen activator
ESC: Embryonic stem cells
NSC/NPC: Neural stem/precursor cells
BBB: Blood-brain barrier
OGD: Oxygen and glucose deprivation
HUVECs: Human umbilical vein endothelial cells
MV: Microvesicles
PBS: Phosphate Buffer Saline
TEM: Transmission electron microscopy
SD: Sprague Dawley
MAP-2: Microtubule associated protein 2
MRI: Magnetic resonance imaging
mNSS: Modified neurological severity score
FBS: Fetal bovine serum
RO: Reoxygenation
ANOVA: One-way analysis of variance
SDF1a: Stromal-derived factor 1
USC-sEV: Urinal stem cell derived sEV
MI: Myocardial infarction
