# Supplementary figures and images for: Small extracellular vesicles secreted by human iPSC-derived MSC enhance angiogenesis through inhibiting STAT3-dependent autophagy in ischemic stroke
Source: Stem Cell Res Ther. 2020 Jul 22;11:313. doi: 10.1186/s13287-020-01834-0 (PMC7374834; doi:10.1186/s13287-020-01834-0)

**Fig. S1**

**A**

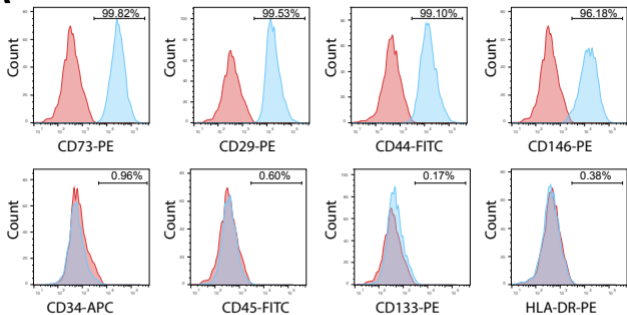

**B**

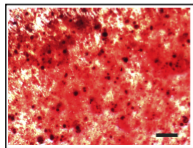

**C**

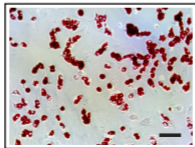

**D**

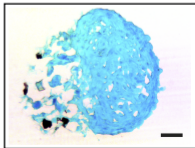

Supplement: Supplementary file 1 — Additional file 1: Figure S1. Phenotypic characteristics of iMSC. (A) Flow cytometry analysis of the surface antigen profile of iMSC. (B-D) Representative images of Alizarin Red staining (B), Oil Red O staining (C), and Toluidine Blue staining (D) for the evaluation of osteogenesis, adipogenesis, and chondrogenesis in iMSC. Scale bar = 25 μm. [file 13287_2020_1834_MOESM1_ESM.pdf]

**Fig. S2**

**A**

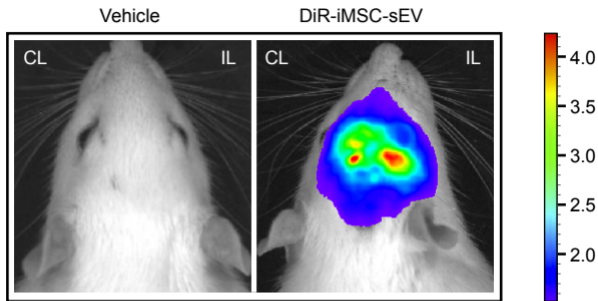

**B**

Experimental design

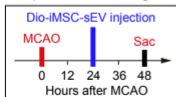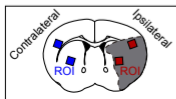

**C**

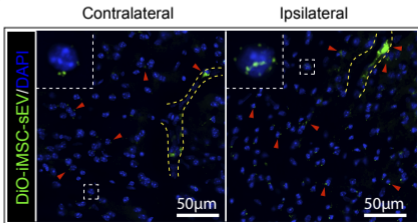

Supplement: Supplementary file 2 — Additional file 2: Figure S2. In vivo uptake of iMSC-sEV after stroke. (A) One single dose of vehicle (PBS, 500 μL) or DiR labeled iMSC-sEV (DiR-iMSC-sEV, 1 × 10^11^ particles in 500 μL PBS) were administered through tail veil injection in rats 4 h after MCAO and images were captured 6 h after administration. Representative fluorescence images of rats brain in the vehicle and DiR-iMSC-sEV group. IL: ipsilateral side. CL: contralateral side. (B-C) Dio labeled iMSC-sEV (Dio-iMSC-sEV) were administered intravenously 24 h after MCAO, and rats were sacrificed 24 h after injection. (B) Illustration of experimental design. (C) Representative images of Dio-iMSC-sEV (green) in the ipsilateral and contralateral side of the brain. Yellow dashed line: outline for blood vessel. Red arrow head: Dio-iMSC-sEV around the nucleus in the cytoplasm. Scale bar = 50 μm. [file 13287_2020_1834_MOESM2_ESM.pdf]

**Fig. S3**

**A**

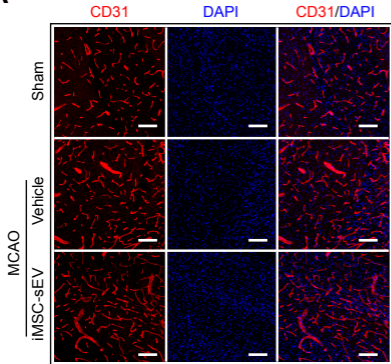

**B**

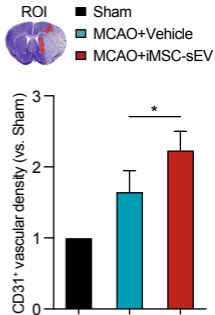

Supplement: Supplementary file 3 — Additional file 3: Figure S3. Treatment of iMSC-sEV increases the blood vessel density after ischemic stroke. CD31 immunofluorescence staining was utilized to evaluate blood vessel density 7 days after MCAO. (A) Representative images of CD31 positive endothelial cells and DAPI staining in the peri-infarct area. Scale bar = 400 μm. (B) ROI and quantification of CD31+ blood vessel density. N = 3–5 per group. Data are presented as mean ± SD. *P<0.05. [file 13287_2020_1834_MOESM3_ESM.pdf]

**Fig. S4**

**A**

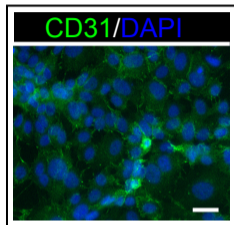

**B**

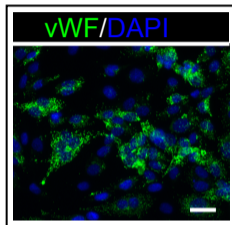

**C**

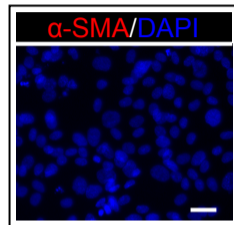

**D**

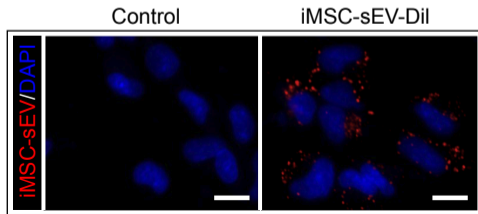

Supplement: Supplementary file 4 — Additional file 4: Figure S4. Identification of HUVECs and in vitro uptake of iMSC-sEV by HUVECs. (A-C) Representative immunofluorescence images of CD31 (green), vWF (green), and α-SMA (red) in HUVECs. Scale bar =100 μm. (D) Representative immunofluorescence images of HUVECs cultured with Dil labeled iMSC-sEV (red) or Dil alone (control). The Dil-labeled iMSC-sEV were visible in the perinuclear region of recipient cells. HUVECs in the control group showed no fluorescence signal. Scale bar =30 μm. [file 13287_2020_1834_MOESM4_ESM.pdf]

# Fig. S5

**A**

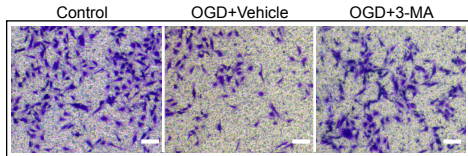

**B**

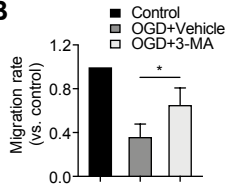

**C**

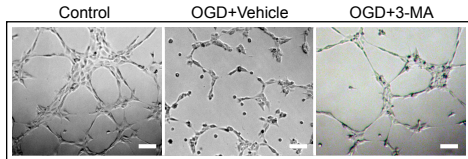

**D**

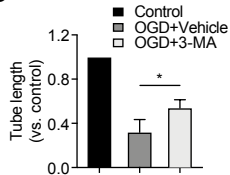

Supplement: Supplementary file 5 — Additional file 5: Figure S5. Inhibition of autophagy decreases migration and tube formation in HUVECs after OGD. HUVECs were challenged with 8 h OGD with the addition of 3-MA (5 mM) or vehicle (PBS) and migration and tube formation were analyzed 24 h after reoxygenation. HUVECs cultured under the normoxia condition without treatment were set as control. (A) Representative images of crystal violet staining in the transwell assay. Scale bar = 25 μm. (B) Quantification analysis of migration rate normalized to control group. N = 3–5 per group. (G) Representative images of the tube formation assay. Scale bar = 25 μm. (H) Quantification analysis of the tube length normalized to control group. N = 3–5 per group. Data are presented as mean ± SD. *P<0.05. [file 13287_2020_1834_MOESM5_ESM.pdf]

# Fig. S6

**A**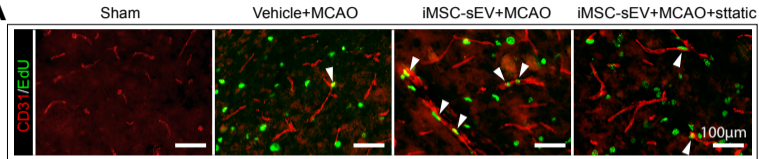**B**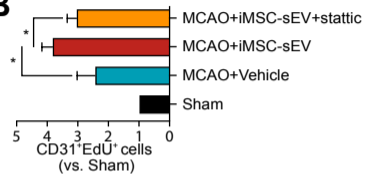**C**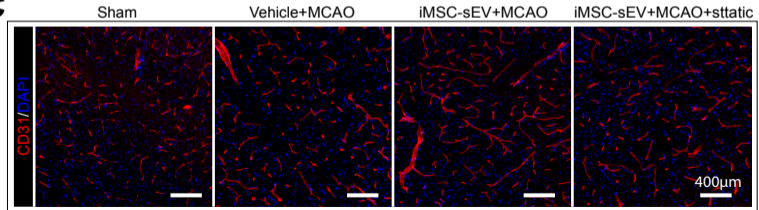**D**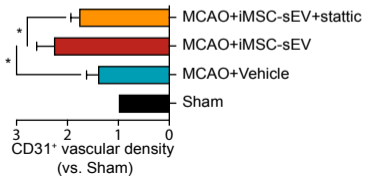

Supplement: Supplementary file 6 — Additional file 6: Figure S6. Stat3 inactivation inhibites iMSC-sEV-induced angiogenesis. (A-D) Rats were intravenously treated with vehicle (500ul, PBS), iMSC-sEV (1 × 10^11^ particles in 500 μL PBS), or iMSC-sEV with stattic (3.75 mg/kg) 4 h after MCAO, EdU was i.p. injected at day 3, 5, and 7 to label proliferated cells. (A) Representative images of CD31+EdU+ endothelial cells in the peri-infarct area. Arrow head, double-labeled proliferated cells. Scale bar = 100 μm. (B) Quantification of CD31+EdU+ endothelial cells normalized to that in sham group. N = 3–5 per group. (C) Representative of CD31+DAPI+ endothelial cells in the peri-infarct zone. Scale bar = 400 μm. (D) Quantification of CD31+ vascular density normalized to that in Sham group. N = 3–5 per group. [file 13287_2020_1834_MOESM6_ESM.pdf]
